# Suppression of NADPH Oxidase Activity May Slow the Expansion of Osteolytic Bone Metastases

**DOI:** 10.3390/healthcare4030060

**Published:** 2016-08-25

**Authors:** Mark F. McCarty, James DiNicolantonio

**Affiliations:** Catalytic Longevity, 7831 Rush Rose Dr, Apt 316, Carlsbad, CA 92009, USA; jjdinicol@gmail.com

**Keywords:** lysophosphatidic acid, osteolysis, NADPH oxidase, TGF-beta, phycocyanin, statins

## Abstract

Lysophosphatidic acid (LPA), generated in the microenvironment of cancer cells, can drive the proliferation, invasion, and migration of cancer cells by activating G protein-coupled LPA receptors. Moreover, in cancer cells that have metastasized to bone, LPA signaling can promote osteolysis by inducing cancer cell production of cytokines, such as IL-6 and IL-8, which can stimulate osteoblasts to secrete RANKL, a key promoter of osteoclastogenesis. Indeed, in cancers prone to metastasize to bone, LPA appears to be a major driver of the expansion of osteolytic bone metastases. Activation of NADPH oxidase has been shown to play a mediating role in the signaling pathways by which LPA, as well as RANKL, promote osteolysis. In addition, there is reason to suspect that Nox4 activation is a mediator of the feed-forward mechanism whereby release of TGF-beta from bone matrix by osteolysis promotes expression of PTHrP in cancer cells, and thereby induces further osteolysis. Hence, measures which can down-regulate NADPH oxidase activity may have potential for slowing the expansion of osteolytic bone metastases in cancer patients. Phycocyanin and high-dose statins may have utility in this regard, and could be contemplated as complements to bisphosphonates or denosumab for the prevention and control of osteolytic lesions. Ingestion of omega-3-rich flaxseed or fish oil may also have potential for controlling osteolysis in cancer patients.

## 1. A Role for Lysosphosphatidic Acid Signaling in Generation of Osteolytic Metastases

Generation of lysophosphatidic acid (LPA) in the microenvironment of cancer cells has emerged as an important driver of the expansion of osteolytic metastases [1]. Many cancer cells express G protein-coupled receptors of the EDG family—LPA1, LPA2, and LPA3—which can be activated by various isoforms of LPA [2]. LPA1 is the most widely expressed LPA receptor, it has the broadest specificity for forms of LPA, and it has received the most research attention to date [3]. Activation of these receptors can promote cellular proliferation and invasiveness, and can also stimulate osteolytic activity in bone [4,5].

Some cancer cells provoke local generation of LPA by triggering aggregation of nearby platelets [6]. Aggregating platelets generate large amounts of lysophosphatidylcholine (LPC), and serum contains modest amounts of a special phospholipase D activity that specifically targets LPC—known as “autotaxin”—which converts LPC to LPA by removing the choline head group [7,8]. Hence, LPA is generated in the microenvironment of aggregated platelets, and can act on cancer cells that have provoked this aggregation. Alternatively, plasma contains meaningful amounts of LPC, reflecting systemic platelet activation, and many cancer cells make and secrete autotaxin, which can convert plasma LPC to LPA near the cell surface [8,9]. Another enzyme produced by some cancer cells that can generate LPA is acylglycerol kinase, which acts on the monoacylglycerol in plasma [10,11]. In prostate cancer patients, the extent to which their cancers expressed autotaxin, but not acylglycerol kinase, correlated positively with risk for biochemical recurrence following surgery [10].

Activation of the EDG family LPA receptors, via heterotrimeric G proteins, promotes activation of diverse signaling pathways, including Akt, RhoA, NF-kappaB, and ERK1/2 [12,13,14]. These pathways can promote proliferation, invasion, and migration, and also, by stimulating the transcriptional activity of AP-1 and NF-kappaB, induce expression of certain cytokines, including IL-6 and IL-8 [6,15,16]. When cancer cells have metastasized to bone, secreted IL-6 and IL-8 can act on neighboring osteoblasts to provoke secretion of RANKL, which in turn can act on macrophages/monocytes to promote their differentiation to osteoclasts [6,9]. The resulting osteolysis tends to release growth factors from the bone matrix—TGF-beta, IGF-I, calcium ions—that provide a further growth stimulus to neighboring cancer cells, in a positive feedback loop [17]. Hence, cancer cells sited in bone that are capable of generating LPA receive a further growth stimulus while causing breakdown of local bone matrix, such that bone tends to be replaced by expanding metastases [18]. Clinical consequences can include severe pain (provoked in part by the acid released during osteolysis), fractures, nerve compression, and hypercalcemia.

## 2. NADPH Oxidase Is a Mediator of both LPA and RANKL Signaling

There is evidence that the signaling pathways stimulated by LPA1, as well as by RANKL, are dependent on activation of NADPH oxidase complexes. Working with PC3 prostate cancer and SKOV3 ovarian cancer, which express LPA1 and are LPA responsive, Daniel and colleagues have shown that the NADPH oxidase inhibitors DPI and apocynin, as well as the antioxidants *N*-acetylcysteine and PEG-catalase, suppress LPA-mediated activation of Akt, ERK, and NF-kappaB [19,20]. Further analysis has indicated that interaction of LPA with LPA1 induces internalization of the complex into early endosomes, following by assemblage and activation of an NADPH oxidase complex affiliated with the endosome that generates hydrogen peroxide within it. This hydrogen peroxide then oxidizes cysteine residues, forming sulfenyl groups, in certain neighboring enzymes, including Akt2 and the tyrosine phosphatase PTP1B. This oxidizing activity presumably is a prerequisite for the downstream activation of LPA1’s key targets, since NADPH oxidase inhibitors, as well as catalase and *N*-acetylcysteine (capable of reversing sulfenic acid formation), block this activation [20]. A study in PC3 cells demonstrates that sequential activation of PLC and PKC mediates LPA-induced NADPH oxidase activation [21].

Moreover, the RANKL-induced promotion of macrophage differentiation into osteoclasts is also dependent on activation of NADPH oxidase activity, as DPI and *N*-acetylcysteine block this differentiation [22,23]. Traf6 and Rac1 are required for NADPH oxidase activation, and the Nox1, Nox2, and Nox4-dependent complexes are involved. Although Nox2 is the form of NADPH oxidase most highly expressed in macrophages, RANKL stimulation downregulates Nox2, and up-regulates Nox1 and 4 [24,25].

## 3. A Role for Nox4 in TGF-beta-Driven Osteolysis

NADPH oxidase activity―specifically, Nox4―may play a role in an additional prominent mechanism, whereby bone metastases promote osteolysis. TGFbeta, released from degraded bone matrix, can induce cancer cells to produce parathyroid hormone-related peptide (PTHrP) [26,27,28,29,30]. This is turn can stimulate osteoblasts to produce RANKL, and suppress their production of the RANKL antagonist osteoprotegerin-thereby activating osteoclastogenesis [31,32,33,34]. Hence, this represents a feed forward mechanism whereby osteolysis can promote further osteolysis. There is reason to suspect that Nox4 induction and activation is required for the efficient induction of PTHrP by TGF-beta. TGF-beta signaling typically entails induction and activation of Nox4 [35,36,37,38].

The mechanism by which TGF-beta induces PTHrP in MDA-MB-231 breast cancer cells has been partially defined [30]. Activation of SMAD signaling, as well as of p38 MAP kinase, collaborate in inducing transcription of the PTHrP gene; the authors refer to p38 as “a new target for osteolytic therapy”. In other contexts, activation of p38 MAP kinase by TGF-beta has been shown to be dependent on Nox4 activation [39,40,41,42]. For example, in TGF-beta-treated mouse embryo fibroblasts, knock-down of Nox4 suppresses p38 MAP kinase activation [39]. This reflects the fact that Nox4 activity is capable of inhibiting MAPK phosphatase 1 (MKP-1) by reversibly oxidizing a key cysteine group in this enzyme. Within the nucleus, MKP-1 functions to dephosphorylate and thereby inactivate the p38 and JNK MAP kinases, and hence its inhibition by Nox4 activity up-regulates p38 MAP kinase signaling [39]. In fibroblasts treated with TGF-beta, Nox4 translocated to the nucleus, and MKP-1 activity was reduced by about 50%.

Hence, there appear to be at least 3 signaling pathways involved in LPA-stimulated osteolysis—those triggered by LPA, RANKL, and TGF-beta—in which NADPH oxidase activation plays a mediating role. It therefore seems reasonable to propose that measures capable of safely down-regulating NADPH oxidase activation or activity could have potential for slowing the expansion of LPA-driven bone metastases.

## 4. Potential Utility of Phycocyanin and High-Dose Statins as NADPH Oxidase Inhibitors

The profound antioxidant activity of the free bilirubin generated within cells by heme oxygenase activity has been traced to inhibition of NADPH oxidase complexes; the isoform-specificity of this effect requires further clarification, although Nox2 and Nox4 appeared to be targeted [43,44,45,46,47]. The fact that elevated serum levels of free bilirubin are associated in prospective epidemiology with a range of favorable health outcomes [48,49,50,51,52,53]—most notably, that individuals with Gilbert syndrome enjoy a 50% reduction in total age-adjusted mortality [54]—suggests that moderate systemic down-regulation of NADPH oxidase activity can be safe and indeed protective in various respects.

Phycocyanin, a key protein in cyanobacteria such as spirulina (constituting up to 20% of its dry mass), and also produced in some eukaryotic algae, is noted for its outstanding antioxidant and anti-inflammatory activity in rodent and cell culture studies; this activity appears to stem from its covalently-attached chromophore, phycocyanobilin (PhyCB) [55,56]. PhyCB is a metabolite of biliverdin, and within cells biliverdin reductase activity can convert it to phycocyanorubin, very similar in structure to bilirubin [57]. Indeed, exposure of human cells to either biliverdin or PhyCB results in dose-dependent inhibition of NADPH oxidase activity, likely mediated by bilirubin and phycocyanorubin, respectively [56,58]. Hence, it has been proposed that the antioxidant activity of orally or parenterally administered phycocyanin reflects, at least in large part, the ability of phycocyanobilin to mimic the physiological antioxidant activity of free bilirubin [56]. In aggregate, these considerations suggest that oral administration of spirulina, phycocyanin, or PhyCB-enriched spirulina extracts may have potential for slowing the expansion of osteolytic bone metastases that is driven by LPA generation. Although few studies to date have evaluated the impact of dietary spirulina on cancer progression in rodents, one recent study reported a 60% reduction in tumor growth rate when nude mice transplanted with a human pancreatic adenocarcinoma were fed a spirulina-enriched diet [59]—likely reflecting a role for constitutive NADPH oxidase activation in driving proliferation and promoting survival of this cancer [60,61,62,63].

Additionally, it is known that, in relatively high doses, statins can suppress activation of certain NADPH oxidase complexes by antagonizing the isoprenylation of Rac1 [64,65,66]. This may explain why, in mice injected with the MDA-MB-231 mammary cancer cell line, concurrent simvastatin treatment notably decreased the areas of osteolysis induced by bone metastases [67]. Nonetheless, statins can influence cellular function in numerous ways, and other mechanisms conceivably could have played a role in this effect.

## 5. Complementary Measures for Suppressing Osteolysis 

Such measures could presumably be used in tandem with anti-osteolytic drugs—bisphosphonates and monoclonal antibodies targeting RANKL (e.g., denosumab)—currently used to slow growth of osteolytic bone metastases [68]. These can provide important pain relief, likely reflecting a role for acid production during osteolysis in triggering pain [69,70,71,72]. A meta-analysis of placebo-controlled trials with zoledronic acid found that, in cancer patients with rapid bone turnover at baseline (NTX > 100 nmol/mmol creatine in urine), use of zoledronic acid was associated with a 31% reduction in mortality risk [73]. Radium-223, although not directly anti-osteolytic, suppresses expansion of bone metastases by killing cancer cells resident in bone [74]. Platelet stabilizing agents have potential for diminishing the production of LPA in the microenvironment of cancer cells [6]. In this regard, an analysis of multi-center controlled trials assessing the effects of daily low-dose aspirin has found that such therapy decreases risk for metastasis formation in patients with pre-existing cancer [75]. This study did not report separately on bone metastases. A more recent meta-analysis confirms this finding [76].

A diet rich in omega-3 fatty acids may also have potential for suppressing cancer-induced osteolysis. Epidemiology has linked increased omega-3 intake to increased bone density [77,78,79]. In ovariectomized rats, diets high in flaxseed or fish oil favorably impact bone density by inhibiting osteolysis [80,81,82,83]. Moreover, in transgenic (fat-1) mice capable of converting omega-6 fats to omega-3s, the adverse impact of ovariectomy on bone density is blunted [84]. Similarly, supplementation with flaxseed (30 g daily) was found to decrease serum levels of the osteolysis marker NTx in healthy humans; a marker for bone formation was not changed [85]. In postmenopausal breast cancer survivors receiving aromatase inhibitor therapy, 4 g daily of fish omega-3s (EPA+DHA) likewise inhibited osteolysis [86]. In vitro, RANKL-stimulated conversion of monocytes to osteoclasts is inhibited by DHA and, intriguingly, arachidonic acid as well [87,88,89,90]. These findings may be rationalized by the recent discovery that DHA, arachidonic acid, and alpha-linolenic acid act as ligands for the farnesoid X receptor (FXR), with binding affinities in the low micromolar range; they also promote the transcription of some but not all gene targets of FXR. FXR agonists have been shown to oppose osteoclastogenesis in vitro [91,92]. The possibility that ample intakes of flaxseed or fish oil could counter cancer-induced osteolysis via FXR agonism merits study in rodents. A diet rich in fish oil or in DHA per se inhibits the formation of bone metastases in nude mice given intracardiac injections of MDA-MB-231 breast cancer cells; to what extent suppression of metastatic seeding in the bone contributes to this effect is not clear [93,94]. In addition to its anti-osteolytic potential, ample intakes of flaxseed can exert anti-proliferative effects on some breast and prostate cancers—cancers known for their propensity to metastasize to bone [95,96]. This anti-proliferative effect has been documented clinically in patients with pre-surgical breast or prostate cancer asked to ingest 30 g of flaxseed daily [97,98].

It should be noted that basal production of IL-8 is elevated in many cancers that are osteotrophic, and, not surprisingly, such cancers are prone to induce osteolytic metastases [99,100,101]. In women with breast cancer, plasma levels of IL-8 were found to be about twice as high in women who had bone metastases, as compared to women without such metastases; in addition, within the whole group, IL-8 levels correlated strongly and positively with serum levels of the osteolysis marker NTx [101]. When nude mice received intra-tibial injections of a breast cancer cell line producing ample amounts of IL-8, all of the mice developed large osteolytic bone lesions; when such mice in addition were treated with an IL-8-neutralizing monoclonal antibody, 83% of the mice remained free of osteolytic lesions [101]. Hence, IL-8-targeting monoclonal antibodies may have potential as a clinical strategy for controlling osteolytic bone metastases.

## 6. Conclusions

In cancers prone to metastasize to bone, generation of LPA in the microenvironment of cancer cells can boost their capacity to stimulate osteolysis, thereby enabling the expansion of bone metastases. This mechanism is now suspected to play a key role in the spread of many osteotropic cancers. An analysis of the signaling mechanisms whereby LPA evokes this effect—including downstream activation of TGF-beta liberated from bone matrix—reveals at least three points (downstream from LPA, RANKL, and TGF-beta receptors) at which stimulation of NADPH oxidase activity plays a mediating role. Hence, measures which can safely down-regulate NADPH oxidase activation or activity may often have utility for slowing the expansion of bone metastases. By mimicking the physiological antioxidant role of free bilirubin, the PhyCB chromophore of spirulina may have potential as a nutraceutical inhibitor of NAPDH oxidase activity. High-dose statins likewise can inhibit NAPDH oxidase activity, by suppressing Rac isoprenylation. These considerations suggest that dietary administration of spirulina or of PhyCB-enriched spirulina extracts should be studied in rodent models of osteolytic bone metastases. Simvastatin has already demonstrated an anti-osteolytic effect in nude mice bearing human breast cancer. These measures, if proven to have efficacy, likely could be used to complement currently employed strategies for controlling bone metastases, such as bisphosphonates, denosumab, and radium-223. Platelet-stabilizing agents, omega-3 fats, and monoclonal antibodies targeting IL-8 may also have anti-osteolytic potential in oncology.
